# Delivery strategies for optimizing uptake of contraceptives among adolescents aged 15–19 years in Nsanje District, Malawi

**DOI:** 10.1186/s12978-020-01065-9

**Published:** 2021-01-20

**Authors:** Andrew Kondaine Makwinja, Zione Mchikaya Maida, Alinane Linda Nyondo-Mipando

**Affiliations:** 1 Department of Health Systems and Policy, College of Medicine, School of Public Health and Family Medicine, University of Malawi, Chichiri, Private Bag 360, Blantyre, Malawi; 2 College of Medicine, African Centre of Excellence in Public Health and Herbal Medicine (ACEPHEM), University of Malawi, Blantyre, Malawi; 3 Medecins Sans Frontieres-Belgium Malawi Mission, Blantyre, Malawi; 4 Ministry of Health, Nsanje District Hospital, Nsanje, Malawi

**Keywords:** Contraceptives, Adolescents, Strategies, Optimizing, Delivery

## Abstract

**Background:**

Despite documented benefits of contraceptives, uptake among young people aged 20–24 years is high compared to adolescents aged 15–19 years in Malawi. As the world’s population of 15–19-year-olds continues to grow the need to meet the increasing demand for contraceptive services and information that address adolescent-specific needs cannot be underestimated. To inform Sexual and Reproductive health services for the youth, we explored strategies for optimizing uptake of contraceptives among this age group.

**Methods:**

An exploratory qualitative cross-sectional study was conducted at Nsanje District Hospital and Nyamadzere Community Day Secondary School guided by Social-Ecological Framework to understand strategies that may optimize the uptake of contraceptives among adolescents aged 15–19. Nsanje district was purposively selected based on the reason that it is the second district in Malawi with the highest rate of adolescent childbearing of girls aged 15–19 years. We conducted a Focus Group Discussion (FGD) with 9 traditional leaders, 11 Key Informant Interviews (KIIs) with health workers, 20 In-depth Interviews (IDIs) with 12 adolescents, 4 teachers, and 4 parents. All data were digitally recorded, transcribed verbatim into English. The data was analyzed and managed using deductive thematic analysis guided by Social-Ecological Framework.

**Results:**

Adolescents suggested accessing contraceptives from local drug stores, pharmacies and hospitals at a health system level and through Youth Centres, clubs, and corners at a Community level. There is a need to ensure a continuous supply of various kinds of contraceptives and the presence of youth-friendly health care workers in the specified areas.

**Conclusion:**

There is no one way of delivering contraceptives to adolescents. Multiple avenues existent at the health facility and community could be leveraged to optimize delivery and uptake of contraceptives in a manner that is not intimidating to an adolescent while involving key stakeholders.

## Plain English summary

Documented evidence shows that contraceptive use prevents pregnancy and childbirth complications which are the leading causes of death among 15 to 19-year-old girls globally. However, the design and implementation of contraceptive service delivery to adolescents among this age group are poorly understood. Therefore, we conducted a study to understand delivery strategies for optimizing uptake of contraceptives among adolescents aged 15–19 years in the rural district of Nsanje, Malawi. We found that adolescents aged 15–19 years need to have strategies through various people and places to optimize the uptake of contraceptives. Hence, collaboration and partnerships between different actors are important to optimize the uptake of contraceptives among adolescents among this age group.

## Background

Despite the documented benefits of contraception, World Health Organization (WHO) in 2015 estimated that the global unmet need for contraceptives among adolescents aged 15–19 years is 23 million and that 50% of pregnancies among this age group are unintended [1]. In sub-Saharan Africa (SSA), contraceptive use among adolescents aged 15–19 years in 2014 ranged 21–42% with an unmet need of 53–64% among unmarried while for the married, contraceptive use was at 8–36% with the unmet need of 16–62% [2]. In Malawi, the use of contraceptives among adolescents aged 15–19 years is markedly lower than the age group 20–24 years. Youths aged 15–19 years used contraceptives at a rate of 25.2% compared to 74.8% in the age group 20–24 years [3].

Although adolescents aged 15–19 years old contribute 23% of global maternal mortality in low and middle-income countries, efforts to prevent the occurrence of teenage pregnancies have been suboptimal and otherwise neglected [4]. Hence, adolescents have been identified as a priority group for preventing maternal deaths globally [1]. Evidence shows that closing the unmet need for modern contraception of women aged 15–19 would reduce unintended pregnancies among this age-group by 6 million annually [5]. The closure will avert 2.1 million unplanned births, 3.2 million abortions, and 5600 maternal deaths [5]. Therefore, increasing the use of contraception among adolescents aged 15–19 years is in tandem with global goals [4].

Several strategies have been employed for adolescents to access contraceptives, such as peer education [6, 7], consumer engagement [6, 8], mass media [7, 9, 10], community-based approaches [11, 12], school-based approaches [13, 14], Comprehensive Sexuality Education [15–18] and youth centers [17, 19]. A study conducted in Malawi advocated for more effective strategies to address the family planning needs of youths [20]. Most of the studies reported insights from parents, adolescents, and health care workers, these include Youth Friendly Health Services, static and outreach youth clubs as well as community awareness conducted by public hospitals, improving counseling services, integrating family planning services and education within school curricula [20–22]. Given that insight from gatekeepers such as community or traditional leaders on optimizing adolescent contraceptive uptake is limited [3, 20, 22], this study explored strategies to optimize uptake of contraceptives among adolescents age 15–19 years old in Nsanje district, Malawi.

## Conceptual framework

This study was guided by the Social-Ecological Framework [23]. The framework recognizes the complex social and environmental system in which individuals exist and how the concentrically larger systems in which they regularly move affect individual behavior at interpersonal, community, organization, and policy levels [23, 24]. Therefore, we used this framework because it allowed identifying strategies from critical areas where decisions on behavior are influenced at an individual (knowledge, attitudes, behaviors), interpersonal (families, friends, and social networks), community (relationships between organizations), institution (organizations and social systems) and policy (national state, local laws) level.

## Methods

### Study design and setting

An exploratory qualitative cross-sectional study was conducted from September to October 2019 in Nsanje district, Malawi [25]. We conducted a Focus Group Discussions (FGDs) among traditional leaders, Key Informant Interviews (KIIs) among health workers, In-depth Interviews (IDIs) among adolescents, teachers, and parents (Table 1). We conducted the study in Nsanje because it has the highest rates of childbearing in adolescents with 38.8% of women and 41.1% of girls (aged 15–19 years) respectively having entered motherhood [27]. YFHS and sexuality education in schools through life skills subjects are the only strategies available for young people to access contraceptives.

**Table 1:** Summary of methods

Sample	Method	Purpose	Sample characteristics	Sample size
Adolescents	In-depth interviews	To understand a condition, experience, or event from a personal perspective	They were categorized into three; school going, out-of-school, and those attending YFHS and were four in each category	12
Teachers	In-depth interviews	To understand a condition, experience, or event from a personal perspective	These were Secondary School teachers and were included in the study as they are the ones responsible for providing sexuality education	4
Parents	In-depth interviews	To understand a condition, experience, or event from a personal perspective	These are responsible for advice and custodian of adolescents upbringing	4
Traditional Leaders	Focus group discussions	To investigate beliefs, attitudes, and concepts of normative behavior	These are influential people and custodians of power who controls the dos and don’ts of the people necessary for maintaining accepted and expected cultural and moral behavior in the community	1 with 9 traditional Leaders
Health Care Workers	Key informant interviews	To explore background information or an institutional perspective	These are professionals such as nurses and clinicians providing YFHS and Family Planning at hospital and Community or primary level	11

### Participant recruitment

We drew a purposive sample of 40 participants based on their age, expert knowledge, and responsibility [28]. (Table 1). The participants included; Adolescents aged 15–19 years, Parents, Health Care Workers working in YFHS and Family Planning, teachers and traditional leaders. The Adolescents were: still in school, out of school, attending YFHS, married or unmarried, and willing to participate in the study with parental consent. The parents selected were recruited if they had children within the age band of 15–19 years. We determined which Health Care Workers from YFHS and Family Planning would be included with assistance from their facility In-charge. All teachers included either taught Life skills or Biology subjects and were identified with the assistance of the head teacher. We recruited the traditional leaders with assistance from the Health Surveillance Assistant.

### Data collection

The PI and Research Assistants collected the data using Interview guides developed from the study objectives and Social-Ecological Framework. All interviews were conducted in private rooms provided by the head teacher at time convenient to the participant. FGDs were facilitated by AKM and ZMM. We ensured data credibility by employing persistent inquiry using probes and to ensure that questions have been responded adequately [30]. After each face to face interview or group discussion the responses were summarized and participants were able to confirm that responses were correctly recorded to ensure quality and trustworthiness of the data [31]. All IDIs, KIIs, and FGDs were conducted in Chichewa, digitally recorded, transcribed, and translated into English. All audios were identified by a number to ensure anonymity and data were stored in a computer protected by a password.

### Data analysis

Interviews were transcribed and translated into English. Data were analyzed using thematic analysis described by Braun and Clarke [32]. Initial interview audios and transcripts were listened to by AKM and ALNM and agreed on an analysis plan. Then codes were deductively generated from the Social-Ecological Framework and study objectives. A coding framework was developed and applied during thematic analysis which involved searching across the transcripts to find repeated patterns and associations on emerging themes [32] focusing on strategies for optimizing contraceptive uptake. Finally, codes were organized under recurring themes of which themes were interpreted by repeated reading of transcripts by AKM. Further, the themes generated were verified against digital recordings and reviewed and discussed with ALNM.

### Ethical approval

Ethical approval was obtained from the College of Medicine Research and Ethics Committee (COMREC P.08/19/2779), and institutional permission was granted by Nsanje District Health and Education offices as well as the Nyamadzere Community Day Secondary School Head teacher. Participation in the study was voluntary and written informed consent was obtained from all study participants before the interview and FGDs. Parental Consent and Adolescent assent were obtained from all participants aged below 18 years per regulations that govern research in Malawi.

## Results

### Demographic characteristics of traditional leaders

Nine participants participated in the FGD. Of these, six were males and three were females. In terms of tribe six were Sena, two Mang’anja, and one Yao. In terms of religion, eight were Pentecostals and one Muslim. Seven participants were educated at the secondary school level, and all the participants were self-employed. The median age of the participants was 41. Eight participants were able to read and no participant received training or orientation on adolescent sexual and reproductive health and YFHS.

### Demographic characteristics of health care workers

Of the eleven key informants, six were males and five were females. Two of the participants were educated to secondary school level and nine to college level. In terms of occupation, three were Nurse Midwife Technicians, two were Nurse Midwife Technicians with Community Health specialization, two were Health Surveillance Assistants, two were Registered Nurse Midwives and two were Clinicians. One Registered Nurse Midwife was the District’s Family Planning Coordinator and One Clinician was the District’s YFHS Coordinator. Two of the respondents had worked for less than 4 years while four had worked for 5–8 years and five for 10–25 years. The median age for key informants was 33.

### Demographic characteristics of teachers

Four teachers participated in the IDIs. Two of the respondents were females and the other two were males. Two of the respondents were married, one was widowed, and one single. In terms of religion two were Catholics, one was Presbyterian and one belonged to the Pentecostal church. Two of the respondents had worked for 13–15 years, one worked for 9 years, and one for 5 years. The median age was 40. No respondent attended training or orientation on adolescent sexual and reproductive health and YFHS.

### Demographic characteristics of parents

Four parents participated in IDIs. Two of the respondents were females and the other two were males. Three of the participants were married and one was widowed. All the four belonged to Pentecostal churches and two of the respondents were educated to the primary school level, one was educated to secondary level and one did not attend formal education. Three were self-employed as farmers and one was a government employee. The median age was 54.5 years. All respondents never heard about adolescent sexual and reproductive health and YFHS before the interviews.

### Demographic characteristics of adolescents

Twelve adolescents participated in IDIs. They were categorized into three; school going, out-of-school, and those attending YFHS and were four in each category. Nine respondents were Pentecostals while two were Presbyterian and one was Adventist. The median age of adolescents was 18 years.

There were two females and two were males in the school going category, and one was in form 2 and the other 3 were in form 3 and all were single in terms of marital status. There were four females in the out-of-school category, one was separated from her partner, one never married and two were married. All of the four out-of-school respondents had a child each. There were two females and two males in the YFHS attendees group. Of these 4, two were school going and the other two were secondary school dropouts and they were all single.

### Proposed strategies for optimizing uptake of contraceptives among adolescents 15–19 years

Participants recommended several strategies for delivering contraceptives among adolescents. The avenues varied from Health system based avenues that could be privately owned pharmacies to community-based spaces. All respondents indicated that adolescents were reluctant to access contraceptives when they felt health care workers at those facilities were likely to disclose this information to their family members.

## Health system based delivery points

### Local pharmacy, drug store, and hospital

The local pharmacy, drug store, and hospital were preferred spaces for delivering contraceptives as suggested by participants from all categories with variations in terms of preference on where to get contraceptives. An adolescent in the out-of-school category prefers that contraceptives be accessed at the nearest pharmacy or drug store because of convenience and potential reduction in impeding factors.

“I think they have to be going to a pharmacy nearby”. (19-year-old, Out of school adolescent_1).

When queried regarding the affordability of contraceptives, she further said that adolescents should get contraceptives from community health care workers especially those who may not be financially able to do so.

“They should use doctors in the villages”. (19-year-old, Out of school adolescent_1).

This was also shared by one parent who stated of covert access to contraceptives for their children.

“Today we see that other health workers are approached secretly by parents to have their children injected contraceptive at their homes they don’t want their children to be seen”. (50 years old, Male parent).

Notably, the health system requires strengthening for it to effectively deliver contraceptives amongst the youth in the specified areas. This could be done by ensuring that the health facilities are well resourced with contraceptives of various types. Participants suggested that contraceptive services to adolescents need to be uninterrupted to avert demotivating adolescents from accessing services.

“We have to make sure all the methods are readily available to avoid frustrations when they find that we do not have we need to be very open and do not discourage them other methods we need to be going for their choices this is not helpful”. (Community Health Nurse_2).

Contraceptive service delivery should be strengthened by ensuring that facilities have a conducive environment for adolescents by having a department for adolescent health that provides all the services needed. Some youths report to the facility in School Uniforms hence needing privacy which can be achieved if they have designated spaces.

“There should be a special room to give all health services in the room for youths where they can come at any time to access what they want because even if you give them special day and time it is difficult they forget the days….apart from that recreation centers in their areas where they go and play they can access contraceptives there and information”. (Community Health Worker_1).

Besides, the health system should invest in having health workers that are trained in YFHS delivery who also able to provide integrated adolescent health services for comprehensive health coverage. Further, the respondents felt that to strengthen the delivery of contraceptives in societies where there is a stronger emphasis on traditional beliefs, bylaws and penalties for teen pregnancies may optimize contraceptive use. It was suggested that these penalties and bylaws should be agreed upon and enforced by traditional leaders in collaboration with the health sector. Some respondents in the KIIs, FGDs, and IDIs suggested that the introduction of bylaws will promote delivery and use of contraceptives among adolescents to avert teenage pregnancies.

“Traditional leaders need to make bylaws so that in their villages teen pregnancies should not happen”. (Community Health Nurses_2).

“We need to make it an agreement with health workers at the villages that we will introduce penalties and you as well at this hospital those coming with teen pregnancies they have to take a letter from the chief to ensure that they have paid the penalty in that way this will encourage them to use contraceptives”. (Male Traditional Leader7_FGD).

## Community-Based delivery centres

### Youth corners

Some respondents in KIIs suggested that there should be Youth Corners which are placed away from the hospital where adolescents access any service regarding their health.

“Like here in Nsanje we don’t have what we call Youth corners where they can get everything and this a big problem in Nsanje….. these are special rooms constructed separately away from people and at a secret place that one has no special day youths go there and find everything there I mean any health service there …”. (Clinician_2).

### Youth club

A youth club is another outlet where adolescents will be comfortable to access contraceptives. Youth clubs are held within a community and school setting.

“Contraceptives should be accessed through youth clubs since youths are shy accessing contraceptives in the hospitals”. (Adolescent 3_YFHS).

It was asserted that having a youth club at school will be a point of contact with health workers and students where they could offer support.

“There should be learners as youths themselves, teachers and one as a patron and once a month authorities have to visit them and monitor and support them”. (Clinician_2).

However, this proposal was regarded as selective as not all students will be members of the youth club hence the best is for health workers to come and meet every student at the general assembly and that learners should belong to youth clubs in the community where they can express themselves freely.

“In a school set up, there is a social gap between learners and teachers the learner does not open up so we should channel this to where learners are coming from like communities”. (Teacher 1_Male).

Respondents also suggested that measures to strengthen attendance to youth clubs could include support from chiefs who would ensure that adolescents within the age of 15–19 years are attending.

“We have to be conducting meetings together with health workers to encourage and support the parents as a chief I have to be working together with the chair of the group and check the register to see which child is not attending the youths groups and make a follow up with parents ….leaving it for youths themselves things will not work”. (Male Traditional Leader6_FGD).

“…We will be doing meetings with them and we will inform their parents that if adolescents are not attending youth groups, they will be excluded from the benefits which others will have**”. (**Female Traditional Leader9_FGD).

Respondents also noted that it was important to have both male and female health workers servicing the youth clubs because some adolescents may not express themselves adequately when the health worker is of the opposite sex.

“…for the provision of contraceptives, some may be shy when they see that the one giving them is a male when they are females because these are sensitive issues I think health workers who will be coming should be two a female and male to fully assist everyone”. (Male Traditional Leader2_FGD).

Schools have programmes called “mother care groups” which need strengthening to effectively deliver and promote contraceptives.

“There are mother care groups there they can be using these groups to sensitize learners they have to be identifying the grownups and teach them about contraceptive use”. (Nurse_1).

### Youth centres

Key informants suggested that there should be Youth Centers or recreation centers for adolescents to have fun and this is where an opportunity to reach them with contraceptives is provided.

“Youth centers in the hospital and outside the hospital need to have open days to provide all things”. (Community Health worker_1).

However, the respondents in IDIs suggested that Youth centers need to be operated by health workers.

**“……..**for the government to implement and sustain it they need to work for hand in handwork community nurse who conducts mobile clinics”. (Nurse_1).

## Discussion

This study outlines delivery strategies for optimizing uptake of contraceptives among adolescents aged 15–19 years. Apart from providing perspectives of parents, adolescents, and health care workers regarding strategies for delivering contraceptives to optimize uptake of contraceptives, findings of this study make an extension to what previous studies have reported by providing perspectives of community or traditional leaders and teachers as well as strategies for strengthening the delivery of contraceptives among adolescents aged 15–19 years.

## Contraceptive delivery strategies for adolescents among 15–19-year-olds

### Local pharmacy, drug store, and hospital

According to the study findings, adolescents prefer to access contraceptives at the nearest hospital, pharmacy, or drug store. The finding is consistent with findings reported by Chandra-Mouli in 2014 and USAID in 2015 where they highlighted that there are various places adolescents prefer to access contraceptives other than the hospital [33, 34]. A study done by Radovich et al. in 2018 reported that young women obtained contraceptives from limited-capacity, private providers compared with older women [35]. They further highlighted that this is the reason adolescents use short acting contraceptive methods than long ones. A possible explanation for this is that adolescents want to access contraceptives in private, a place where they feel that confidentiality and privacy are guaranteed [34]. This implies that adolescents prefer to access contraceptives from a variety of places especially places convenient to them eventually making delivery of contraceptives to adolescents more complex. Thus, informing adolescent sexual and reproductive health programs and experts to consider a wide range of places where adolescents can access contraceptives [33]. Therefore, there is a need to make local pharmacies and drug stores adolescent-friendly [34]. More importantly, this may be realized if policymakers and providers partner with private pharmacy and drug store owners for the adolescents to have wide access to contraceptives including long-term contraceptive methods [36]. The findings build on the argument by FHI360 Ethiopia 2004 report which reported that government and donors support private commercial ventures such as private franchise clinics and contraceptive social marketing that provide reproductive health services, though these were not necessarily targeted to youth [37]. In situation of barriers with access to a private pharmacy or drug store such as affordability, this study suggested parents’ covert access to contraceptives for their children through Community Health Care workers. Therefore, this informs future research to understand parents’ covert access to contraceptives for their children through Community Health Workers in Nsanje, Malawi.

### Youth Centered Services-Youth Corners, clubs and centers

Youth specific spaces such as Youth Clubs, Youth Centers, and Corners in the community as strategies for optimal uptake of contraceptives among adolescents provide a platform for the social network which supports adolescents to access contraceptives [38]. One of the spaces the current study reported is a Youth Friendly Corner which is a private space staffed by dedicated young volunteers where youth access counseling and referrals for sexual and reproductive health services without adult interaction [39]. Similarly, Youth Friendly Corners were reported to be a huge source of information on contraceptives for young people in Zambia and Zimbabwe [39, 40]. Unlike Youth clubs and Centers which combine recreation activities, Youth Corners are dedicated to providing SRH services [39]. Consistent with our findings, an earlier study in 3 Malawian districts reported youth clubs as a strategy for optimizing uptake of contraceptives among young people [20]. These are places where young people meet in the community and participate in 'life skills' training, health education sessions, and recreational activities [41]. Our study suggests that health workers make school visits to address all learners on SRH issues to mitigate the social gap between teachers and learners. Youth Centers are meeting points and “one-stop shops” which are intended to be a friendly, safe, and non-clinical environment where SRH services and information is provided alongside social services such as recreational activities or internet cafes’ [17]. A possible explanation for this suggestion by the participants is that these places which operate on a static or mobile basis are convenient to adolescents and provide an alternative for contraceptive access to adolescents as they shun hospitals [34]. Thus, underscoring the need for program implementers and policymakers to establish more outlets beyond the health facility for expanded adolescent contraceptive access in the district and policies that support such services respectively. In terms of performance, separate studies conducted in Machinga, Malawi, and a review by Zuurmond in 2012 reported low uptake of SRH services in Youth clubs and centers [19, 22]. Apart from health workers providing SRH stocks to youth clubs as alluded to earlier [22], the current study asserts for the availability of young health care workers, male and female to serve and operate the youth clubs and centers to mitigate challenges with gender and age and traditional leaders to have a leading role of youth clubs, centers and corners in their respective communities. Furthermore, use of peers as deliverers of contraceptives to curb fear was also illustrated in other studies where involvement and buy-in from adolescents was underscored as an important factor in ensuring access and use of contraceptives [6, 34, 36, 42]. This calls for policy makers and implementers to incorporate adolescents in the programming and implementation of activities aimed at improving uptake of contraceptives among adolescents.

### Strengths and limitations of the study

One strength of this study is that the study used the Social-Ecological Framework which helped the study to capture the views of people from implementers of the strategies such as health care workers, community leaders, parents, teachers, and adolescents which are users of contraceptives. This allowed a wide gathering of data on key thematic areas. Therefore, the study has managed to bring an understanding of areas that influence the uptake of contraceptives among adolescents hence the findings can be used to inform further research, policy, and practice. However, one limitation to the study is that the findings are from a qualitative sample and are confined to one setting, Nsanje district, and may not reflect the views of other locations in Nsanje district and beyond.

## Conclusion

To optimize uptake of contraceptives among adolescents aged 15–19 years we need interventions that consider not only individuals but also their environment. When talking of environment we must consider the range of health professionals and facilities that provide contraceptive advice and information at the individual, community, and institutional level. Also, our study found the need for interventions to strengthen the delivery of contraceptives at individual and community levels to influence adolescents to use the people and places for delivering contraceptives. Furthermore, interventions at an institutional level such as providing adolescent health separate from adults through adolescent centered units (one-stop center) may contain health worker challenges in prioritizing and negotiating with adolescents when delivering health services. Nevertheless, in culturally contextualized society, bylaws, and penalties for teen pregnancies by traditional leaders in collaboration with the health sector may optimize uptake of contraceptives. Partnerships with local private drug stores may optimize uptake by removing access obstacles such as cost.

## Data Availability

Datasets used are available from corresponding author upon request.

## Abbreviations

HCW: Health care workers
IDIs: In-depth interviews
KIIs: Key informant interviews
FCDs: Focus group discussions
YFHS: Youth friendly health services
SRH: Sexual reproductive health
